# Principles of Nanoparticle Design for Genome Editing in Plants

**DOI:** 10.3389/fgeed.2022.846624

**Published:** 2022-03-07

**Authors:** Pushkal Sharma, Tedrick Thomas Salim Lew

**Affiliations:** ^1^ Department of Chemical Engineering, Massachusetts Institute of Technology, Cambridge, MA, United States; ^2^ Department of Chemical and Biomolecular Engineering, National University of Singapore, Singapore, Singapore; ^3^ Institute of Materials Research and Engineering, Agency for Science, Technology and Research (A*STAR), Singapore, Singapore

**Keywords:** nanotechnology, gene editing, precision breeding, nanoparticles, CRISPR

## Abstract

Precise plant genome editing technologies have provided new opportunities to accelerate crop improvement and develop more sustainable agricultural systems. In particular, the prokaryote-derived CRISPR platforms allow precise manipulation of the crop genome, enabling the generation of high-yielding and stress-tolerant crop varieties. Nanotechnology has the potential to catalyze the development of a novel molecular toolbox even further by introducing the possibility of a rapid, universal delivery method to edit the plant genome in a species-independent manner. In this Perspective, we highlight how nanoparticles can help unlock the full potential of CRISPR/Cas technology in targeted manipulation of the plant genome to improve agricultural output. We discuss current challenges hampering progress in nanoparticle-enabled plant gene-editing research and application in the field, and highlight how rational nanoparticle design can overcome them. Finally, we examine the implications of the regulatory frameworks and social acceptance for the future of nano-enabled precision breeding in the developing world.

## Introduction

The Green Revolution in the 1960s enabled a steep rise in food security *via* the development of hybrid crops, fertilizers, and institutional mechanisms, benefitting many regions of the world by reducing malnourishment and poverty (Bailey-Serres et al., 2019). However, traditional cross-breeding and mutagenesis to generate desired traits are time-consuming and untargeted, and the crop yield improvement enabled by the Green Revolution has been steadily declining (Tilman et al., 2011; Chen et al., 2019). As a result, the current crop yields are estimated to fall short of the world’s projected population demand by 2050 (Ray et al., 2013). This is exacerbated by ever-increasing abiotic and biotic stresses, limited genetic variation, and increasing resource costs (Bailey-Serres et al., 2019). Fortunately, with improvement in our understanding of underlying metabolic and protein interaction networks in plants, complemented by emerging genetic engineering technologies, it is possible to manipulate plant traits at the genomic level to help address these challenges. Recent progress in transgenic research has led to significant advances in engineering crops for improved yield and stress tolerance. Genome-wide association mapping has shown that single-nucleotide polymorphisms are enough to generate agriculturally-important trait variation in crop plants (Zhao et al., 2011; Hu et al., 2015; Bharat et al., 2020). Pathogen-recognizing receptor genes have been transferred between unrelated plant lineages to confer immunity against pathogenic strains (Boutrot and Zipfel, 2017; Koller et al., 2019), while the introduction of transcription factors from wetland species into staple crops has enabled repression of specific gene clusters to confer submergence tolerance without any yield penalty (Voesenek and Bailey-Serres, 2015; Dar et al., 2018). Plant biomass production has been increased considerably by modulating the chromatin accessibility by demethylase overexpression increasing crop yields (Yu et al., 2021), and manipulation of non-photochemical quenching-related enzymes helped tobacco plants in adjusting to fluctuating light conditions (Kromdijk et al., 2016). The ability to engineer gene expression also enables other applications such as the production of therapeutic compounds at scale (Fausther-Bovendo and Kobinger, 2021), rapid production of vaccines with increased immunogenicity (Fausther-Bovendo and Kobinger, 2021), removal of unwanted metabolites (Padmaja, 1995), or large-scale knockout screens to probe unknown biological pathways (Gaillochet et al., 2020).

Rapid progress in genome editing technologies offers new opportunities to alter plant genome with nucleotide-scale precision for crop improvement. Base editing enzymes can perform point mutagenesis and have been used to generate herbicide-resistant crops (Shimatani et al., 2017; Tian et al., 2018; Zhang et al., 2019a; Bharat et al., 2020). The introduction of a single indel has been used to generate splice variants of the host plant translation factor disrupting its interactions with a viral protein which helped reduce the infection titer under field conditions (Gomez et al., 2019). While sequence-specific nucleases, such as zinc-finger nucleases and transcription activator-like effector nucleases (Wright et al., 2005; Christian et al., 2010), have been used for targeted editing of the plant genome, their application is limited due to the construction complexity of such large nucleases. CRISPR-Cas (clustered regularly interspaced short palindromic repeats/CRISPR-associated protein) is an alternative genome editing technology that relies on DNA-RNA binding for sequence-specific cleavage, offering design simplicity and ease of use at minimal cost (Mali et al., 2013; Koonin et al., 2017). The CRISPR-Cas toolbox is increasingly used to perform such genetic manipulation in plants, enabling gene knockout, base editing, organelle genome editing, and transcriptional regulation precisely in a targeted manner (Zhang et al., 2019b). As alluded earlier, non-native genes and accompanying desirable traits can be introduced into a wide range of plant species using transformation protocols where DNA is delivered into plant cells by either a particle gun or using plant-infecting soil bacterium *Agrobacterium tumefaciens*. However, these conventional methods insert varying copies of DNA at random locations in the host plant genome, leading to a low field performance (Meyer, 1995; Day et al., 2000; Dong and Ronald, 2021). Therefore these methods are usually supplemented by time-consuming regeneration of hundreds of independently transformation plant cells to screen for those with single-copy insertions and optimal phenotype (Mumm and Walters&, 2001). On the other hand, CRISPR-Cas platform has been successfully used for targeted knock-in of marker-free DNA into specific endogenous genomic sites for optimized expression due to the high specificity of Cas nucleases and low off-target base-pairing rates of guide RNAs (Schiml et al., 2014; Zhao et al., 2016). Owing to their unparalleled ability to induce precise nucleotide changes, CRISPR-Cas systems have emerged as a powerful tool to improve crop yields and stress resistance. For example, disrupting *Gn1a*, *DEP1,* and *GS3* genes in rice led to larger grain size and higher yield (Li et al., 2016), targeted mutations in the *MLO* allele produced bread wheat resistant to powdery mildew (Wang et al., 2014), and editing the promoter region of host sucrose transporter genes generated rice lines with broad-spectrum resistance against bacterial blight (Oliva et al., 2019). By inducing targeted nucleotide variations to achieve the desired output, CRISPR has the potential to accelerate the transition from conventional cross-breeding to precision breeding for crop improvement.

**FIGURE 1:**
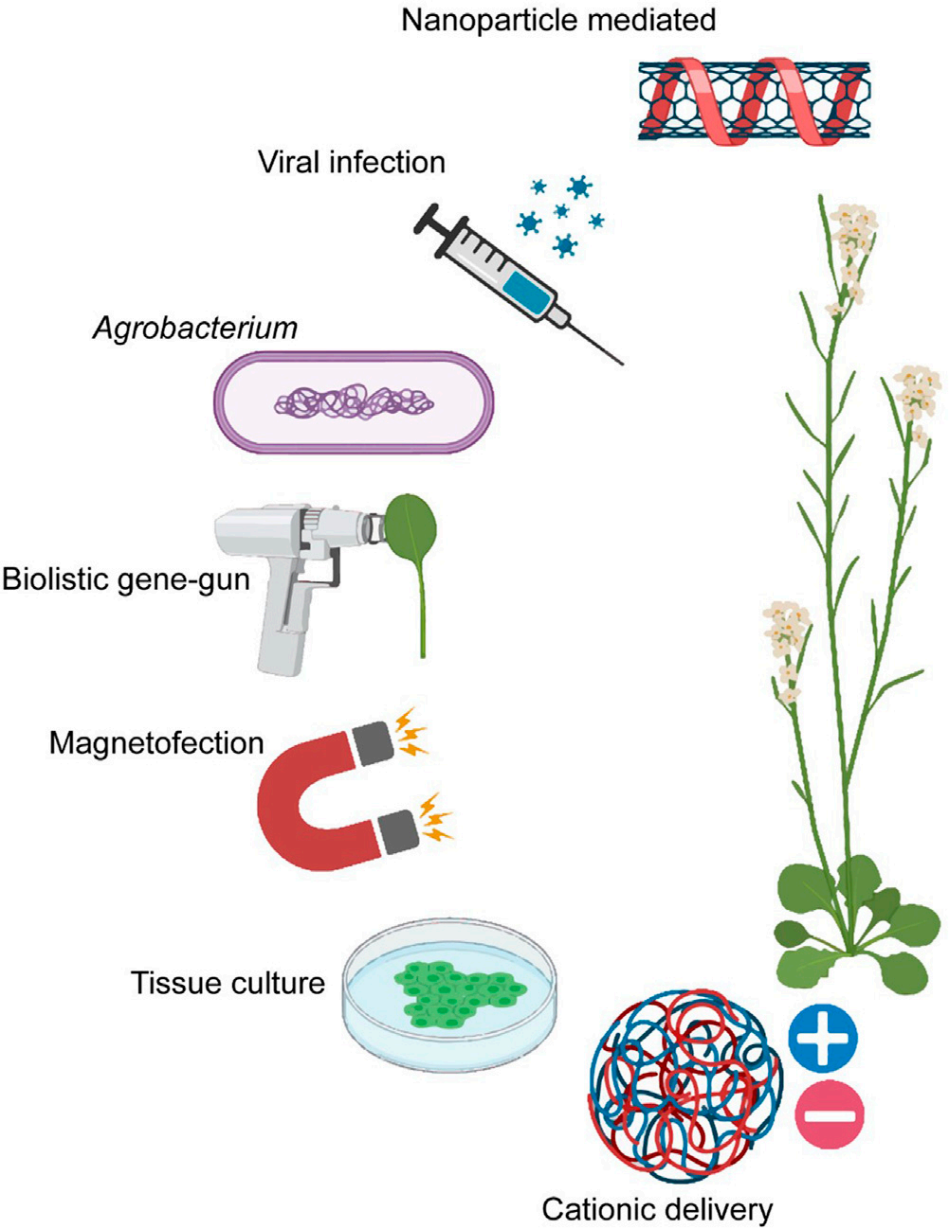
The current modes of cargo delivery that can be employed for CRISPR Cas reagent delivery *in planta*: nanoparticle-mediated (Lew et al., 2020c), viral infection (Ma et al., 2020), Agrobacterium (Zhang et al., 2019c), biolistic gene-gun (Miller et al., 2021), magnetofection (Zhao et al., 2017), tissue culture (Nasti and Voytas, 2021) and cationic delivery (Duan et al., 2021) (Created with BioRender.com).

An essential requisite for applying CRISPR technologies in agriculture is a robust and efficient way of delivering CRISPR reagents into plant cells. They are generally delivered into plant cells as plasmids or RNA-protein complexes by particle bombardment (Klein et al., 1992), *Agrobacterium*-mediated delivery (Zhang et al., 2019c), cationic delivery (Duan et al., 2021), or viral infection (Ma et al., 2020) (Figure 1). These conventional delivery systems are limiting in many ways. *Agrobacterium* or viral vectors apply only to a narrow range of species due to host-range constraints, and their efficiency in transformation efficiency is significantly affected by the plant genotype (Chen et al., 2019). Furthermore, *Agrobacterium*-mediated delivery has yielded low transformation efficiencies (particularly in monocots) and is limited in modulating the amount of delivered donor templates which affects the insertion efficiency and frequency of unintended sequence disruptions (Mao et al., 2019). Alongside, it leads to random genomic integrations *via* nonhomologous recombination, which may disrupt essential genes (SB, 2017) and trigger regulatory concerns due to potential off-target mutations (Jones, 2015). Chemical methods such as cationic polymers are typically used to target protoplasts and thus require the establishment of suspension cells, protoplast isolation, and species-dependent regeneration protocols (Sandhya et al., 2020). Biolistic gene-gun or electroporation delivery is based on the mechanical rupturing of the target tissue by pressurized helium gas but is limited by tissue damage, nonspecific subcellular bombardment sites, low penetration depths, and sporadic editing efficiencies (Ahmar et al., 2021). Current genetic engineering methods also typically target immature, undifferentiated tissues (callus or meristems) and therefore require costly and laborious tissue culture protocols to generate progeny (Cunningham et al., 2018). Transient transformation of CRISPR-Cas reagents is preferred over stable integration due to fewer regulatory restrictions, shorter breeding cycles, and fewer unintended off-target effects (Liang et al., 2017). This remains challenging due to the large cassette, high charge density, and low ionic stability of Cas9 protein, which needs to traverse through the protective plant cell wall. An alternative is first to introduce Cas9 ribonucleoproteins into wall-less protoplasts, but this requires tissue regeneration, which is challenging to use across species, especially for recalcitrant species (Mao et al., 2019). Consequently, an unmet need remains to devise an effective, low-cost, and universal strategy to deliver gene-editing cargo into plant cells.

## Nanotechnology to Address Challeges for Plant Gene Editing

Multiple proof-of-principle studies over the past few years have shown that nanoparticles, in particular carbon nanotubes (CNTs), can be used to deliver nucleic acid-based cargoes to plant species and tissues efficiently in an almost species-independent manner (Demirer et al., 2019; Kwak et al., 2019). Owing to the small size of nanoparticles (typically defined as those with dimensions of less than 500 nm), they have been shown to transport past the plant cell wall and cellular membranes to deliver genetic cargo and to detect biomolecules (Lew et al., 2018; Lew et al., 2020a; Lew et al., 2020b; Lew et al., 2021). Transient expression of exogenous DNA in the chloroplasts and nucleus was demonstrated recently by leaf infiltration of cationic CNTs in mature arugula, spinach, wheat, and cotton, among others (Demirer et al., 2019; Kwak et al., 2019). The comprehensive molecular toolbox of surface chemistry enables facile functionalization of nanoparticles, potentially allowing the conjugation of CRISPR RNA-protein complexes to be protected from degradation, delivered to targeted cellular regions, and finally cleaved from the carrier in a controlled fashion, as has been demonstrated in mammalian systems (Deng et al., 2020; Wei et al., 2020). Direct penetration of pollen surface apertures for transformation could circumvent the need for regeneration from tissue culture, allowing direct production of edited offspring as demonstrated recently with single-walled CNTs (Lew et al., 2020c). Tissue culture and regeneration burden can also be reduced by targeting shoot apical meristem, which are usually inaccessible behind multiple tissue layers and require large penetration depths (Demirer et al., 2021). Layer-by-layer assembly on surface functionalized nanoparticles (Richardson et al., 2016) can potentially stagger Cas9 expression and sgRNA release for maximum transformation efficiency. Loading a whole array of nucleic acids or proteins on the same nanocarrier could simultaneously express multiple regulatory players in a controlled spatiotemporal fashion. This type of gene stacking is a promising approach for improving a complex desirable trait where multiple pathways need to be simultaneously perturbed but is challenging to achieve as genes need to be expressed in a targeted temporal manner which otherwise could lead to undesired pleiotropic effects (Vanhaeren et al., 2016).

## Plant Cellular Barriers For Targeted Nanoparticle Delivery

**FIGURE 2:**
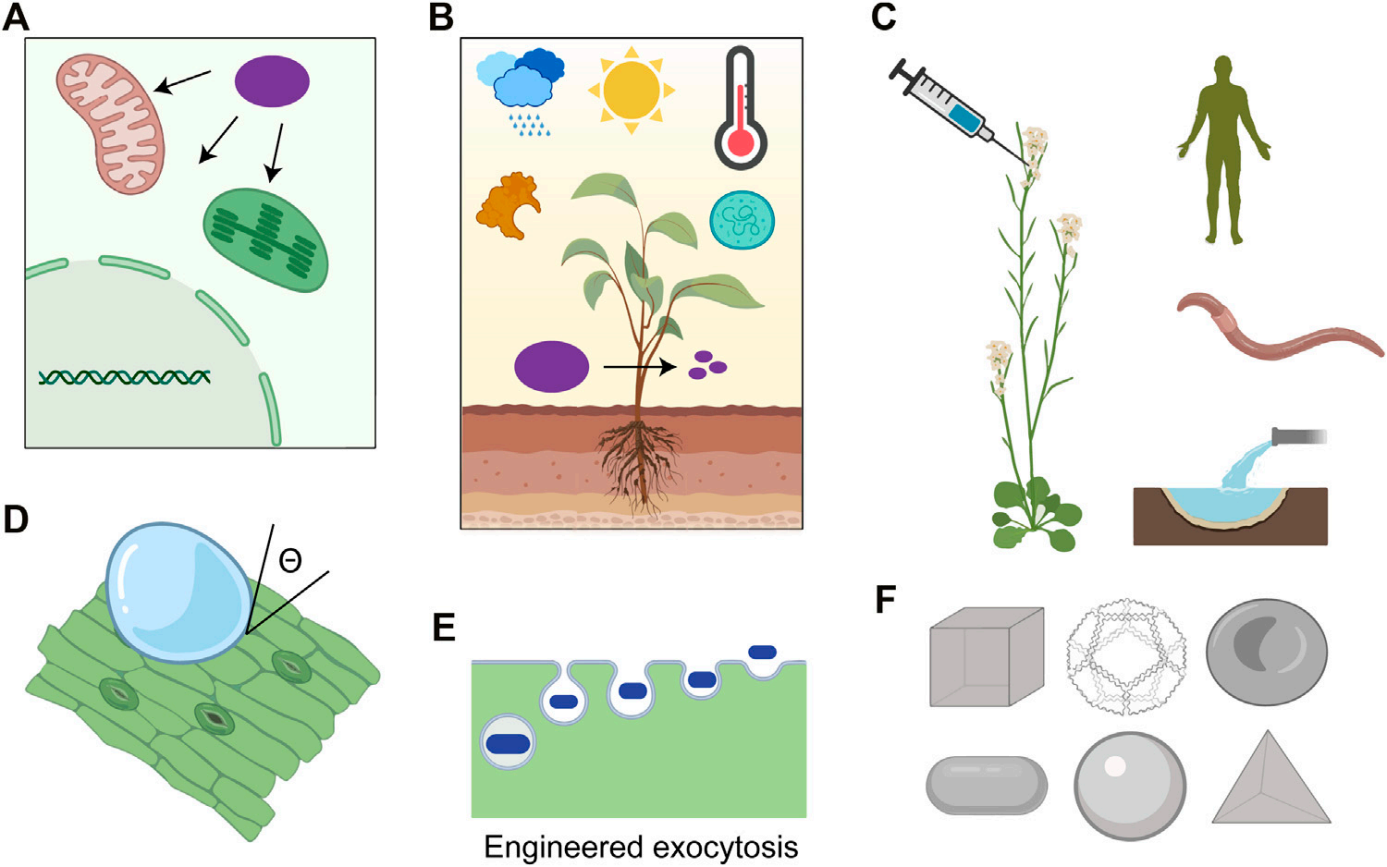
**(A)** Targeting cargo, such as CRISPR reagents (purple), to organelles (green: chloroplast, red: mitochondria, and grey: nucleus) in plants allows the independent genetic engineering of these structures in their native context. **(B)** Nanoparticle design framework should account for the field conditions the cargo (purple) would be exposed to, such as sunlight, extreme temperatures, moisture, nucleases, and microbes. **(C)** Lifecycle assessment of plant nanoparticle gene editing effects on humans, soil biome, and nearby water ecosystems should be investigated. **(D)** Formulation optimization of the nanoparticle suspensiontomaximize wetting (represented by contact angle of suspension with leaf, ϴ) without compromising photosynthesis. **(E)** Exocytosis pathways of nanoparticles from plant cells and their engineering remains unclear. **(F)** Nanofabrication techniques such as DNA origami can be used to study the effects of nanoparticle size, shape, and functionalization on biodistribution and editing efficiencies (Created with BioRender.com).

While there have been successful laboratory demonstrations for nanoparticle trafficking into plant cells in recent years, the general mechanism of nanoparticle transport past the cell wall and the plasma membrane into various subcellular organelles remains unclear (Lew et al., 2020d). This lack of knowledge of nanoparticle interactions with plant cellular membranes has significantly hampered the development of reliable nanoscale tools to deliver gene-editing cargo (Lew et al., 2020a). For example, gene editing reagents can be accurately targeted to the mitochondria to engineer development and stress tolerance (Liberatore et al., 2016). Physical models explaining the distribution of nanoparticles within plant cells have been proposed, but how conjugation of biomolecular cargo alters nanoparticle localization remains understudied (Lew et al., 2018). Spatial control over the nanoparticle subcellular localization will uncover the potential utility of nanoparticles in organelle genome editing and metabolic engineering (Figure 2A) (Saminathan et al., 2021). Previously peptide biorecognition motif conjugations have been used to deliver plasmid DNA into chloroplast and mitochondria of intact plants (Yoshizumi et al., 2018; Thagun et al., 2019), but their use for targeting CRISPR Cas reagents remains unexplored. This is primarily due to the sub-efficient delivery of guide RNA and Cas9 into organelles which can be addressed by the available diversity of functionalization chemistries for peptide-nanoparticle conjugates. Leveraging endogenous receptor-ligand interactions can further allow targeting of loaded nanoparticles to specific cell types or organs in intact plants, as shown recently for *C. elegans* (Chakraborty et al., 2021).

The delivery barriers can be overcome by rationally manipulating nanoparticle design parameters such as size, shape, stiffness, and surface charge. In mammalian cells, nanoparticles averaging ∼100 nm generally undergo long-lasting circulation (Blanco et al., 2015), while nanoparticles with diameters <5 nm undergo rapid renal clearance and are eliminated from the body (Choi et al., 2007). Similarly, geometrical features such as particle orientation, aspect ratio, and curvature affect nanoparticle phagocytosis’s kinetics, leading to the exploration of simple shapes such as cylindrical, ellipsoidal, and discoidal for cancer therapeutics (Dasgupta et al., 2014; Vácha et al., 2011; Champion and Mitragotri, 2006). The switchable surface charge has also been explored in mammalian systems to maximize the cellular uptake of nanoparticles (Yuan et al., 2012) since the negative charge promotes longer circulation times by decreasing nonspecific uptake (Yamamoto et al., 2001) while positive charge prevents cargo degradation within the endosomal compartment (Nel et al., 2009). Mechanical properties such as tensile strength and deformability also promote longer circulation and accumulation (Zhang et al., 2012). Similar counterpart explorations are necessary for plant cells since they differ from mammalian cells in several ways—additional cell wall barrier, porous cuticle, and the diversity of epidermal leaf features such as stomata and trichomes. Such optimization at the nanoparticle design stage will significantly reduce runoff losses to agricultural soils, associated environmental risks (increased exposure or change in exposure routes), and resource intensity (embodied water, energy, and emissions from upstream processing) while increasing genetic transformation efficiencies (Figures 2B,C) (Xi et al., 2021). However, conventional nanofabrication techniques do not allow the exploration of a more diverse shape and size design space. DNA origami is an especially promising branch of nanoparticles that utilizes Watson-Crick base pairing to enable high-yield and monodisperse synthesis of virtually any arbitrary structures with user-defined periodicity, asymmetry, or curvatures (Dietz et al., 2009; Han et al., 2011; Benson et al., 2015). Unique sequence and positions of staples allow nanometer-resolution addressability meaning functional moieties (including proteins and aptamers) can be site-specifically placed at desired locations on the nanoparticle structure, which provides unmatched control of nanoparticle-cell membrane interactions and uptake pathways (Bastings et al., 2018; Dey et al., 2021). The stoichiometry of cargo to scaffold molecules can be modulated to limit the wasteful runoffs and enable exceptional control over CRISPR reagent dosage per individual nanoparticle (Hu et al., 2018). This is accompanied by the ability of these structures to resist enzymatic degradation (Mei et al., 2011), have minimal off-target effects (Lee et al., 2012), and release payloads in response to specific cues with spatial and temporal precision (Li et al., 2018). Many ever-expanding DNA-modification chemistries allow an unparalleled toolkit to explore the opportunity space of physical and chemical properties, (Yang et al., 2015; Stephanopoulos, 2019) thus presenting a versatile platform to investigate nanostructures’ structure-property relationships for precision gene editing.

## Nanoparticle-Enabled Plant Gene Editing At Scale

Plant gene editing at scale remains challenging to realize even in the presence of multiple successful studies showing the use of nanoparticles to deliver a variety of cargoes into intact plants. Most conventional gene-editing methods require regeneration from tissue culture, which remains a significant bottleneck in scaling (Zhang et al., 2020). The requirements for hormones and growth media are not defined for most species for differentiating somatic cells during tissue culture. Further, the cell passaging during culture is mutagenic and requires several months to years (Nasti and Voytas, 2021). Infiltration of nanoparticles into leaves is also currently limited by the obligatory use of puncturing and pressurizing (Demirer et al., 2019). With this method, the ratio of nanoparticles entering the plant to the applied amount is often overlooked, and it remains unclear why specific formulations demonstrate better uptake into the plant systems than the others (Su et al., 2019). Additionally, such a method is not amenable for large-scale applications outside the laboratory. An alternative way to introduce CRISPR cargo is through an aerosol-mediated foliar spray, where gene editing can be performed by spraying carbon nanoparticles similar to fertilizer application. This is especially promising for non-commercial applications such as genome-wide organismal screens in a controlled laboratory setting. Further improvements will require careful optimization of the nanoparticle suspension formulations to increase wettability with leaf surface (smaller contact angle between leaf-suspension interface) and retention on leaf surface under field environment (sunlight, microbes, temperature, pH, nucleases, organic matter, and rain) since leaves are the standard uptake route for nanoparticles in plants. This would also increase the intrinsic nanoparticle activity, i.e., observed transformation efficiencies normalized by the exposed surface area and loaded reagent amount.

Interestingly a wide variety of contact angles have been reported for leaves of different plant species with water (Wang et al., 2015; Kwak et al., 2017) implying that the variations in leaf morphological features may dictate the generalizability of using nanoparticle mediate gene editing across different plant species (Figure 2D). The leaf wettability depends on several factors, including the wax content and structure, trichome density, stomatal aperture and density, leaf water content, and shape of epidermal cells (Wang et al., 2015). Very high wettability of a suspension towards leaves may lead to a significant reduction in photosynthesis rates due to 10,000 fold difference in diffusion rates of carbon dioxide in water than air (Hanba et al., 2004). Surfactants can lower the surface tension of the nanoparticle formulations to increase adhesion and spread on the leaf surface but may denature the attached cargo proteins. Such surfactant molecules are also known to penetrate the cuticle and increase their conductance, increasing the nanoparticle uptake rates (Räsch et al., 2018). This presents a unique opportunity to characterize different plant species and optimize design formulations for maximal intrinsic nanoparticle activity without affecting plant growth rates. A few reports have shown promising results, which stress the importance of unexplored design space of wettability and sustained retention of nanoparticles. Layered double hydroxide clay nanosheets can protect dsRNA cargo from RNase treatment while adhering to the leaf surface even after vigorous rinsing and providing sustained release (Mitter et al., 2017). Deployment of nanoparticles for at-scale plant gene editing is also limited by the technical issues of mass production, such as scalability, batch-batch variability, and reproducible performance under different environmental conditions.

## Environmental and Regulatory Considerations for Nanoparticle-Enabled Plant Gene Editing

Classical genetically modified (GMO) crops are subjected to stringent regulatory frameworks due to the insertion of foreign DNA into the plant genome. In contrast to traditional transgenic approaches, nanoparticle-mediated delivery may enable transgene-free genome-edited crops *via* transient expression of CRISPR reagents. This could potentially circumvent GMO labeling in many countries and substantially lower the cost of regulatory processes associated with genome-edited crops, thus encouraging innovation, affordable access, and commercialization of these crops. However, the regulatory landscapes concerning plants modified with gene-editing technologies are different in various regions of the world. While the European Court of Justice’s ruling to regulate genome-edited crops the same way as conventional GMO could stifle progress in plant genome editing (Zaidi et al., 2019), the rulings by United States and Japan to relax the regulations for genome-edited crops signaled a positive atmosphere which could lead to less restrictive regulatory purviews worldwide (Waltz, 2018).

The knowledge gaps in the understanding of the ecotoxicity, exposure pathways, and lifecycle impact assessments of nanoparticles may make it challenging to navigate the environmental and safety reviews by the regulatory agencies. This is further accompanied by other political, legal, consumer acceptance, economic, business, and ethical challenges about the use of nanoparticles (Yang and Duncan, 2021). Therefore, the question of the safe use of nanoparticles for plant gene editing for non-laboratory settings remains unexplored and will have to be reviewed by the public health agencies, consumers and manufacturers after considering environmental, performance, and economic trade-offs. This will add complexity to the already uncertain regulatory framework surrounding genome-edited crops. Such decision-making can be informed by sentinel field data collection at multiple test sites, though this will require dedicated long-term funding opportunities.

Nanotechnology still constitutes a novel tool to probe the fundamental biology of plants in plant science research by enabling genetic perturbations in a species-independent and efficient manner. While there remains a lack of comprehensive data on their effect on plant transcriptome and metabolic network, it can be addressed by the advent of next-generation omics technologies (such as genomics, transcriptomics, proteomics, and metabolomics), which can capture the molecule-level alterations introduced by these nanoparticles. Such information can then lead to a more advanced design of nanoparticles which may facilitate their rapid degradation, exocytosis, and excretion from plants after genetic cargo delivery, reducing toxicity or contamination concerns (Figures 2E,F). For example, peptide and chemical functionalization of nanoparticles has been used to regulate their exocytosis in mammalian cells (Bartczak et al., 2012; Oh and Park, 2014; Kim et al., 2015; Gravely et al., 2019), and similar strategies may be pursued to regulate nanoparticle fate in the plant cellular environment.

## Conclusion

Nanoparticle-enabled gene-editing techniques have the potential to revolutionize agriculture owing to their ability to transform plants in a species-independent and non-integrating manner. Facile surface chemistry allows versatile modification of nanoparticle physicochemical properties, allowing versatile functionalization to protect and safely deliver gene-editing cargoes into targeted compartments within plant cells. Barriers to the application of nanoparticles in agriculture include low transformation efficiency, *in planta* stability, lack of high-throughput delivery method, as well as understudied fate and exposure of these nanoparticles in the environment. In order to fully harness the potential of nanobiotechnology in agriculture, not only do more research opportunities need to be generated to ensure nanoparticles’ efficacy and safety, but social acceptance and an environment facilitating nanoparticle use in plant biotechnology are also essential. Although the field is still in its infancy, the increasing acceptance and changing regulatory landscape supporting nanoparticle use in healthcare and nanomedicine may signal a promising framework that can be adapted to facilitate the adoption of nanoparticles in agriculture. Nano-enabled precision breeding is predicted to be a powerful weapon against poverty and hunger, and conducive regulatory landscapes and support mechanisms need to be provided to develop this promising field.

## Data Availability

The original contributions presented in the study are included in the article/Supplementary Material, further inquiries can be directed to the corresponding author.
